# Physical activity and exercise in the treatment of depression

**DOI:** 10.3389/fpsyt.2012.00106

**Published:** 2012-12-07

**Authors:** Holly Blake

**Affiliations:** Faculty of Medicine and Health Sciences, Queen's Medical Centre, University of NottinghamNottingham, UK

Mental health problems continue to present a global challenge and contribute significantly to the global burden of human disease (DALYs). Depression is the most common psychiatric disorder and is thought to affect 121 million adults worldwide, and as such was rated as the fourth leading cause of disease burden in 2000 (Moussavi et al., 2007), projected to become the highest cause of disease burden by 2020. Antidepressant drugs are an effective and commonly used treatment for depression in primary care (Arroll et al., 2009), although almost half of those treated do not achieve full remission of their symptoms, and there remains a risk of residual symptoms, relapse/recurrence (Fava and Ruini, 2002). In those patients who do demonstrate improvements in depressive symptoms with antidepressant therapies, a time-lag in the onset of therapeutic effects is frequently reported. Antidepressant drugs are associated with adverse side effects (Agency for Health Research and Quality (AHRQ), 2012) and an increased risk of cardiovascular disease, particularly in those with pre-existing cardiovascular conditions or major cardiovascular risk factors (Waring, 2012). Furthermore, adherence to antidepressant medications is often poor and patients often prematurely discontinue their antidepressant therapy; it has been suggested that approximately 50% of psychiatric patients and 50% of primary care patients are non-adherent when assessed 6-months after the initiation of treatment (Sansone and Sansone, 2012).

Psychological treatments for depression have been recommended in the UK National Institute for Health and Clinical Excellence (NICE) guidelines (NICE, 2009) and are becoming more commonplace for helping to reduce symptoms in depressed adults (Ambresin et al., 2012; Brakemeier and Frase, 2012), with even brief psychosocial interventions showing promise for improving adherence to depression medication treatment in primary care settings (Sirey et al., 2010). However, attendance at psychological intervention sessions can be poor since many depressed adults who may benefit from such treatments choose not to attend mental health clinics due to the perceived stigma of psychological therapies.

As such there has been an increasing interest in the role of alternative interventions for depression. Physical exercise has been proposed as a complementary treatment which may help to improve residual symptoms of depression and prevent relapse (Trivedi et al., 2006). Exercise has been proposed by many as a potential treatment for depression and meta-analysis has demonstrated that effect sizes in intervention studies range from -0.80 to -1.1 (Rethorst et al., 2009). However, the evidence is not always consistent; recent research has shown that that provision of tailored advice and encouragement for physical activity did not improve depression outcome or antidepressant use in depressed adults when compared with usual care (Chalder et al., 2012). Other researchers have failed to find an antidepressant effect of exercise in patients with major depression but have found short term positive effects on physical outcomes, body composition and memory (Krogh et al., 2012). Others have argued that the nature of exercise delivery is an important factor, with exercise of preferred (rather than prescribed) intensity shown to improve psychological, physiological and social outcomes, and exercise participation rates in depressed individuals (Callaghan et al., 2011).

Research findings have been summarized by a recent Cochrane review which reported the findings of 32 randomized controlled trials in which exercise was compared to standard treatment, no treatment or a placebo treatment in adults (aged 18 and over) with depression (Rimer et al., 2012). This review concluded that exercise seems to improve depressive symptoms in people with a diagnosis of depression when compared with no treatment or control intervention, although highlighted that this should be interpreted with caution since the positive effects of exercise were smaller in methodologically robust trials. Similarly, a systematic review found that physical exercise programs obtain clinically relevant outcomes in the treatment of depressive symptoms in depressed older people (>60 years; Blake et al., 2009). Although the positive effects of exercise intervention on depressive symptoms are gaining more clarity, reviews suggest that there are currently insufficient high quality data to determine cost-benefit of exercise intervention in depression (Blake et al., 2009; Rimer et al., 2012). Many intervention studies with depressed populations are hampered by methodological weaknesses and small samples sizes. Further, comparisons between studies are often difficult due to variations in assessment or diagnosis of depression, level of severity of the condition, setting for delivery and size of the sample, outcomes of interest and the nature of the intervention delivered (type, frequency and duration of the intervention).

Despite some inconsistencies in research findings, in the UK, the value of exercise continues to be substantiated by current reports and guidelines which include exercise as a management strategy for depression; NICE guidelines have recommended structured, supervised exercise programs, three times a week (45 min to 1 h) over 10–14 weeks, as a low-intensity Step 2 intervention for mild to moderate depression (NICE, 2009); Scottish Intercollegiate Guidelines Network (SIGN) for non-pharmaceutical management of depression in adults has recommended that structured exercise may be considered as a treatment option for patients with depression (SIGN, 2010); and exercise is specified as a treatment option for people with depression in a report for the National Service Framework for Mental Health (Donaghy and Durward, 2000). This is further substantiated by research which demonstrates that patients also find value in physical activity as an effective treatment for depression (Searle et al., 2011), although their perceptions of potential benefits and barriers to participation vary between individuals.

The relationship between exercise and improved physical and psychological health is very well established in both healthy populations and also in people with long-term conditions, and active lifestyles are generally promoted in all populations where physical activity can be safely undertaken. Depression has been clearly associated with low levels of physical activity (Biddle, 2000; Goodwin, 2003) although this does not necessarily infer causality—there are many reasons why individuals who are depressed may have a more sedentary lifestyle, not least recognizing the effects of depression on motivation to engage in healthy lifestyle behaviors.

We know that physical activity confers positive effects on mental well-being although the exact mechanisms which support this relationship are still poorly understood. Patients with depression have attributed this to a number of subjective benefits including biochemical pathways, and cognitive mechanisms include diversion from negative thinking, and a sense of purpose (Searle et al., 2011). Researchers have attempted to clarify this association and have identified a range of possible explanations. The potential role of the inflammatory response has been highlighted as a key mechanism in understanding the relationship between exercise and mood (Hamer et al., 2012). It has also been proposed that physiological changes associated with exercise including endorphin and monoamine levels, or reduction in the levels of the stress hormone cortisol (Duclos et al., 2003) may exert an influence on mood. Further, a growing body of research on the role of neurogenesis in the etiology and treatment of depression has indicated that exercise may alter neurotransmitter function, and promote growth of the hippocampus which is known to be reduced in depressed populations (Lucassen et al., 2010). Indeed, laboratory studies have shown that the neurogenic response to exercise has been found to be much stronger than the response to antidepressant medications (Marlatt et al., 2010). Whilst researchers continue to investigate the mechanisms for this relationship the fact remains that physical activity is good for physical and mental health and therefore important for all.

The social contact often derived from physical activity may play an important role in the relationship between physical activity or exercise and mood. Social support is known to be important for mental well-being, although early studies with older adults showed that exercise reduces depressive symptoms equally to social contact, with exercise also exerting a broader effect than social contact alone through reducing somatic symptoms (McNeil et al., 1991). Others have shown that physical activity intervention may improve mood and quality of life (QoL) equally to social contact in older adults, although this is yet to be tested in comparison with a “no-contact” control group (Kerse et al., 2010).

The focus on the relationship between QoL and exercise is increasingly evident although there are few well-designed studies which have examined the relationship between physical exercise and QoL in depressed individuals. Improvements in global functionality, depressive and general psychopathological symptoms have been observed in depressed patients who have undertaken a supervised exercise regimen adjunctive to standard therapy with antidepressant drugs, with concomitant improvements in perceived QoL although only in the “physical domain” (Carta et al., 2008). Current trials are including QoL as an important outcome variable in exercise interventions for patients with long-term conditions (e.g., Saxton et al., 2012). In fact, it has been argued that the promotion of exercise should now focus more heavily upon the benefits for QoL than focusing on the physical health benefits which have historically predominated in health promotion efforts (Stevens and Bryan, 2012).

QoL is an important outcome criterion for interventions with depressed patients, particularly since patients with depressive disorders and/or depressive symptoms have been shown to have substantial and long-lasting decrements in multiple domains of functioning and well-being that equal or exceed those of patients with chronic medical illnesses (Hays et al., 1995). Definitions of QoL vary in the literature with some definitions focusing on individuals' perceptions of their health status, whereas other definitions focus on individuals' levels of satisfaction with their health status. However, a commonly cited definition is “a state of well-being that is a composite of two components: (1) the ability to perform everyday activities that reflect physical, psychological, and social well-being and (2) patient satisfaction with levels of functioning and the control of disease and/or treatment-related symptoms” (Gotay et al., 1992). Exercise has been associated with QoL in epidemiological studies, and regular exercise has shown to substantially improve QoL in populations with serious long-term conditions such as cancer (Burnham and Wilcox, 2002), Stroke (Smith and Thompson, 2008) and chronic obstructive pulmonary disease (Emery et al., 1998). However, there is less evidence for exercise improving QoL in disease-free populations. Although QoL has been advocated as either a primary or secondary outcome in health research (Speight and Barendse, 2010), a recent systematic review revealed that very few exercise and depression trials have actually included QoL as an outcome (Schuch et al., 2011).

Largely due to the small number of intervention studies in this area, and methodological weaknesses within published studies, exercise intervention studies have not consistently demonstrated effects of exercise on QoL outcomes (Spirduso and Cronin, 2001; de Vreede et al., 2007), although in studies that *do* show positive effects, exercise dose has been found to be a significant predictor of change in mental and physical aspects of QoL (Martin et al., 2009). However, assessing QoL continues to be challenging, particularly in depressed populations where there may be an overlap in measurement between QoL and psychopathology; depression is known to negatively impact on different aspects of an individual's life and this in turn can result in significant impairments in QoL (Ishak et al., 2012). The influence of depressive symptomatology on QoL scores may therefore invalidate research results (Aigner et al., 2006).

Overall, the evidence suggests that exercise can improve depressive symptoms and this is observed even in those suffering from major depressive disorder (Pilu et al., 2007) who have been shown to benefit more from physical exercise than other psychiatric groups (Tordeurs et al., 2011). Exercise, additionally, may exert a positive influence on QoL, although these benefits are subjective in nature and measurement can be difficult due to methodological concerns. In practice, clinicians may be somewhat hesitant to recommend lifestyle changes to depressed patients since they may lack the motivation to exercise. This may be hampered further by public media coverage of negative trial findings which can amplify the difficulties in persuading patients with depression to take exercise (Trueland, 2012). However, the magnitude of the known health benefits of exercise for all mean that researchers have proposed this as a “first-line therapy” in all patients (Nahas and Sheikh, 2011) where prescription should be tailored to patients' current level of activity, preferred type and intensity of activity.
